# Toll-like receptor 4 deficiency: Smaller infarcts, but nogain in function

**DOI:** 10.1186/1472-6793-7-5

**Published:** 2007-06-25

**Authors:** Se-Chan Kim, Alexander Ghanem, Heidi Stapel, Klaus Tiemann, Pascal Knuefermann, Andreas Hoeft, Rainer Meyer, Christian Grohé, Anne A Knowlton, Georg Baumgarten

**Affiliations:** 1 Department of Anesthesiology and Intensive Care Medicine, University of Bonn, Germany; 2 Department of Internal Medicine, University of Bonn, Germany; 3 Institute of Physiology, University of Bonn, Germany; 4 Department of Internal Medicine, University of Bonn, Germany; 5 Division of Cardiovascular Medicine, University of California, Davis, USA

## Abstract

**Backgound:**

It has been reported that Toll-like receptor 4 (TLR4) deficiency reduces infarct size after myocardial ischemia/reperfusion (MI/R). However, measurement of MI/R injury was limited and did not include cardiac **function**. In a chronic closed-chest model we assessed whether cardiac **function **is preserved in TLR4-deficient mice (C3H/HeJ) following MI/R, and whether myocardial and systemic cytokine expression differed compared to wild type (WT).

**Results:**

Infarct size (IS) in C3H/HeJ assessed by TTC staining after 60 min ischemia and 24h reperfusion was significantly smaller than in WT. Despite a smaller infarct size, echocardiography showed no functional difference between C3H/HeJ and WT. Left-ventricular developed pressure measured with a left-ventricular catheter was lower in C3H/HeJ (63.0 ± 4.2 mmHg vs. 77.9 ± 1.7 mmHg in WT, p < 0.05). Serum cytokine levels and myocardial IL-6 were higher in WT than in C3H/HeJ (p < 0.05). C3H/HeJ MI/R showed increased myocardial IL-1β and IL-6 expression compared to their respective shams (p < 0.05), indicating TLR4-independent cytokine activation due to MI/R.

**Conclusion:**

These results demonstrate that, although a mutant TLR4 signaling cascade reduces myocardial IS and serum cytokine levels, it **does not preserve myocardial function**. The change in inflammatory response, secondary to a non-functional TLR-4 receptor, may contribute to the observed dichotomy between infarct size and function in the TLR-4 mutant mouse.

## Background

Activation of Toll-like receptor 4 (TLR4) initiates a sequential activation of IL-1 receptor-associated kinases (IRAKs), tumor necrosis factor (TNF)-receptor-associated factor 6 (TRAF6), nuclear factor κB inducing kinase (NIK), and the IκB kinase complex (IKKs). IKK activation induces phosphorylation and degradation of IκB followed by activation of nuclear factor κB (NFκB) [1]. NFκB induces the activation of proinflammatory cytokines, especially TNF and IL-1β, which are well-known for their cardiodepressive effects [2].

Innate immunity is involved in myocardial ischemia/reperfusion (MI/R) injury, specifically through activation of TLR4, which was originally proposed to solely recognize lipopolysaccharide (LPS) [3]. Other cell products than LPS, may be released from injured tissue and are potential ligands of TLR4. Among these ligands are heat shock proteins (HSP) 60 and 70, and matrix proteins, such as fibrinogen, fibronectin and hyaluronic acid [4-7]. Thus, these ligands, which can be released by necrosis, may compound reperfusion injury by activation of the TLR4 signaling pathway. Consistent with this hypothesis, Oyama et al. demonstrated that in TLR4-deficient mice infarct size and signs of inflammatory response were significantly decreased after MI/R [8]. Moreover, Chong et al. showed in the same strain that myocardial mRNA expression of the cytokines IL-1β and IL-6 was significantly lower after MI/R, when compared to their wild type littermates. Surprisingly, MI/R did not induce significant TNF expression in either WT or TLR-4 deficient mice [9]. In contrast, other studies have shown that MI/R increases myocardial TNF [2]. However, it remains unclear whether subsequent effects of TLR4-activation during MI/R injury are beneficial or deleterious for cardiac function. Recent studies have demonstrated that TLR4 mediates survival in cardiac myocytes and that LPS treatment could be cardioprotective in certain settings. [10-13].

It has not been shown that a reduction in infarct size in the TLR4-deficient mice results in improved hemodynamics and cardiac function. The expression of cytokines after MI/R is unclear with conflicting reports [2,9]. Therefore, we investigated the importance of TLR4 for cardiac function in TLR4-deficient (C3H/HeJ) and wild type mice (WT, C3H/HeN) in a murine model of chronic, closed-chest MI/R. Furthermore, we measured myocardial and serum cytokine levels to determine if locally and systemically expressed TNF, IL-1β and IL-6 are regulated via TLR4 following MI/R.

## Results

### Infarct Size

**Figure 1:**
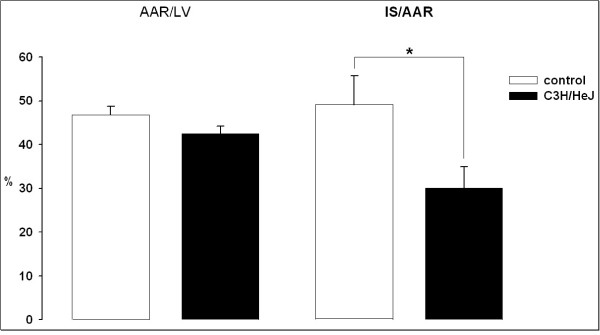
Area at risk as a percentage of left ventricle (AAR/LV) and myocardial infarct size as a percentage of area at risk (IS/AAR) in both experimental groups assessed by TTC staining. There was no significant difference in area at risk of left ventricle indicating equal position of LAD occlusion. Infarct size is significantly smaller in C3H/HeJ mice (WT: n = 10, C3H/HeJ: n = 15; *p < 0.05).

Infarct size in C3H/HeJ mice was significantly reduced at 30.1 ± 4.8% of the area at risk (AAR; figure 1) compared to WT (49.1 ± 6.6%). AAR as a percentage of the left ventricle was 46.8 ± 1.9% and 42.5 ± 1.7% in WT (n = 10) and in C3H/HeJ mice (n = 15), respectively (p = ns, figure 1).

### Cardiac Function

**Figure 2:**
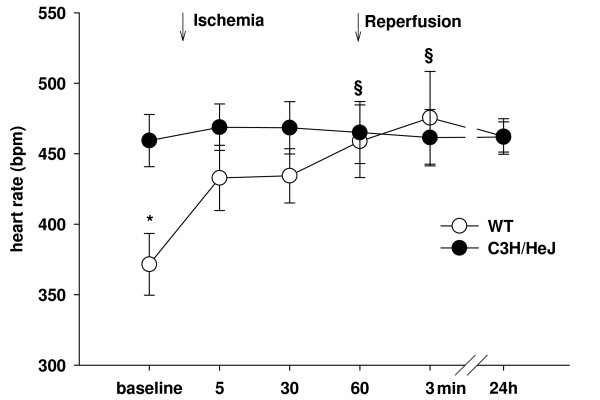
Heart rate before and during MI/R measured with ECG (WT: n = 12, C3H/HeJ: n = 12, *p < 0.05 vs C3H/HeJ, §p < 0.05 vs baseline).

**Figure 3:**
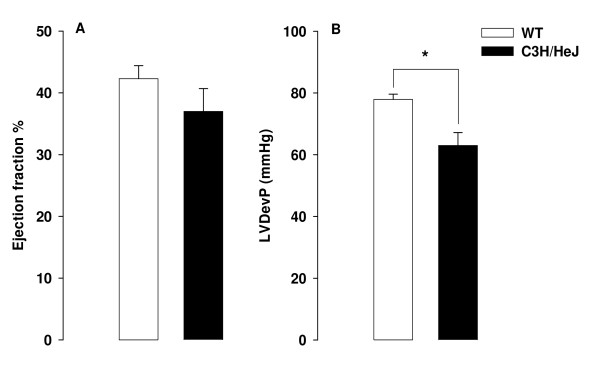
Cardiac function determined by ejection fraction (EF%) and LV developed pressure (LVDevP), measured with echocardiography (WT: n = 6, C3H/HeJ: n = 6) and LV-catheter (WT: n = 6, C3H/HeJ: n = 3, *p < 0.05).

In WT, the heart rate increased from 372 ± 22 min^-1 ^before ischemia to 459 ± 26 min^-1 ^(p < 0.05) after 60 min of ischemia (figure 2). Unexpectedly, heart rate in C3H/HeJ was higher at baseline, 459 ± 18 min^-1^, and did not change throughout the protocol (465 ± 22 min^-1 ^after 60 min of ischemia). Echocardiography detected no difference in ejection fraction (EF) between sham-operated WT (n = 14) and C3H/HeJ (n = 7) mice (62.6 ± 2.2% and 56.2 ± 3.4%, respectively, p = ns). 24 h following MI/R (figure 3A), EF was similarly reduced in WT (42.3 ± 2.1%, n = 6) and C3H/HeJ mice (37.0 ± 3.7%, n = 6, p = ns), reflecting moderate infarction.

Immediately after echocardiography, hemodynamic parameters were assessed (figure 3B). In three C3H/HeJ MI/R mice, catheterization was technically not feasible. There was no significant difference between groups with respect to heart rate 24 h after MI/R. Contractility (+dp/dt_max_) and relaxation (- dp/dt_min_) showed a tendency towards impaired LV function in C3H/HeJ mice (table 1). Although both groups of mice had similar EF by echocardiography, a marked difference was seen in LV developed pressure (63.0 ± 4.2 mmHg in C3H/HeJ, n = 3 vs 77.9 ± 1.7 mmHg in WT, n = 6, p < 0.05, table 1).

**Table 1:** Hemodynamic and echocardiographic parameters

**Strain**	**Heart rate (bpm)**	**LVDevP (mmHg)**	**+dp/dt_max _(mmHg·s^-1^)**	**- dp/dt_min _(mmHg·s^-1^)**	**ESV (μL)**	**EDV (μL)**
WT	588.4 ± 33.6	77.9 ± 1.7*	7887.5 ± 317.3	-5554.9 ± 402.6	32.4± 3.4	55.6± 4.5
C3H/HeJ	587.7 ± 11.4	63.0 ± 4.2	6651.0 ± 1383.0	-6117.2 ± 1129.4	33.8 ± 5.8	52.2 ± 6.1

Hemodynamic measurements (WT n = 6, C3H/HeJ: n = 3) and echocardiography (WT n = 6, C3H/HeJ: n = 6) were performed 24h after MI/R. Hemodynamic parameters showed no significant difference for contractility (+ dp/dt_max_) and relaxation (- dp/dt_min_). However, left-ventricular developed pressure (LVDevP) was significantly higher in WT. End-systolic left-ventricular volume (ESV) and end-diastolic left-ventricular volume (EDV) showed no difference. *p < 0.05.

### Heart weight: Post-Ischemia Myocardial Edema

Differences in cytokine expression in response to ischemia could contribute to differences in myocardial wall edema post-ischemia, and effect function. Post-ischemic myocardial wall edema can contribute to stiffness of the heart and potentially protect ejection fraction [14]. Therefore, we measured heart dry and wet weight for post MI/R myocardial edema, as well as heart to body weight ratio. No significant differences were found between C3H/HeJ (n = 6) and WT MI/R (n = 5) and corresponding sham groups (table 2).

**Table 2:** Heart wet/dry weights, heart/body weight ratio

**Treatment**	**strain**	**Heart wet/dry**	**Heart wet/body weight (%)**
**MI/R**	**WT (n = 5)**	3.5 ± 0.5	0.34 ± 0.01
	**C3H/HeJ (n = 6)**	3.7 ± 0.4	0.32 ± 0.01
**Sham**	**WT (n = 5)**	4.0 ± 0.4	0.33 ± 0.01
	**C3H/HeJ (n = 6)**	3.5 ± 0.5	0.30 ± 0.02

Heart wet to dry weight ratio and heart wet to body weights. p = ns

### Myocardial TNF, IL-1β and IL-6

**Figure 4:**
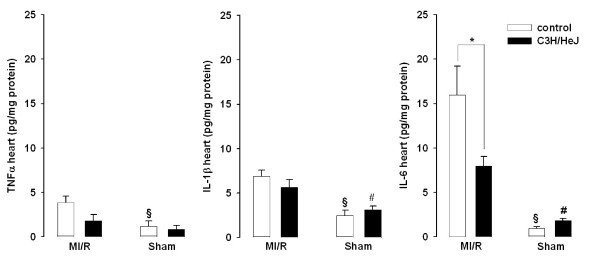
Myocardial protein expression of TNF, IL-1β and IL-6 in WT and C3H/HeJ mice following MI/R and 2 hours of reperfusion or sham operation determined with ELISA. TNF and IL-1β levels were increased, but not signficantly in WT mice compared with C3H/HeJ. IL-6 was significantly higher in WT mice (16.0 ± 3.2 pg/mg protein vs. 8.0 ± 1.1 pg/mg protein). *p < 0.05 vs C3H/HeJ MI/R, § p < 0.05 vs WT MI/R, # p < 0.05 vs C3H/HeJ MI/R.

Activation of TLR4 induces production of the pro-inflammatory cytokines TNF, IL-1β and IL-6. We measured myocardial cytokine expression after two hours of reperfusion (figure 4). As might be expected, WT myocardial IL-6 levels were higher compared to both WT sham and to C3H/HeJ after MI/R. However, there was no difference in IL-1β levels in WT vs. C3H/HeJ mice after MI/R. Likewise, TNF did not differ between the two groups after MI/R. Interestingly, C3H/HeJ with MI/R had higher myocardial IL-1β and IL-6, but not TNF levels, than their respective sham group (p < 0.05), (table 3A). This suggests TLR4-independent induction of IL-1β and IL-6 after MI/R. For WT, all three cytokine levels were higher after MI/R vs. sham (p < 0.05)

**Table 3:** Myocardial and serum TNF, IL-1β and IL-6 expression

**A**				
**Treatment**	**strain**	**TNF heart**	**IL-1β Heart**	**IL-6 heart**

**MI/R**	**WT**	3.9 ± 0.7*^#^	6.9 ± 0.7*^#^	16.0 ± 3.2*^#‡^
	**C3H/HeJ**	1.8 ± 0.7	5.7 ± 0.8*	8.0 ± 1.1*
**Sham**	**WT**	1.2 ± 0.6	2.4 ± 0.6	0.9 ± 0.2
	**C3H/HeJ**	0.9 ± 0.4	3.1 ± 0.4	1.9 ± 0.2

**B**				

**Treatment**	**strain**	**TNF serum**	**IL-1β Serum**	**IL-6 serum**

**MI/R**	**WT**	491.1 ± 138.6^†^	314.9 ± 76.3^†^	1890.6 ± 78.6^†^
	**C3H/HeJ**	41.2 ± 9.6	85.29 ± 32.2	1178.1 ± 314.2^¥^
**Sham**	**WT**	17.3 ± 7.2	17.3 ± 7.2	17.3 ± 7.2
	**C3H/HeJ**	10.6 ± 3.2	26.0 ± 5.6	74.5 ± 23.1

**A **Myocardial and **B **serum protein levels of TNF, IL-1β and IL-6 in WT and C3H/HeJ mice following MI/R and 2 hours of reperfusion or sham operation determined with ELISA. All serum cytokines were significantly higher in WT mice which underwent MI/R compared to C3H/HeJ. n = 5 for each group, *p < 0.05 vs C3H/HeJ sham, #p < 0.05 vs. WT sham, ^‡ ^p < 0.05 vs C3H/HeJ MI/R, ^† ^p < 0.05 vs all groups, ^¥ ^p < 0.05 vs. WT sham and C3H/HeJ sham

### Serum Levels of TNF, IL-1β and IL-6

**Figure 5:**
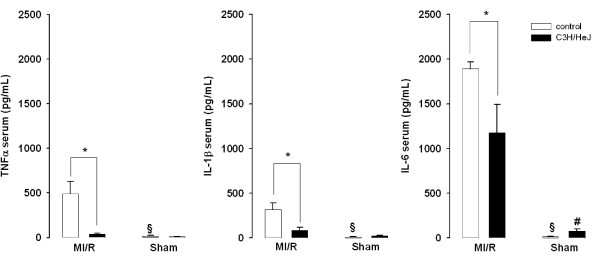
Serum protein levels of TNF, IL-1β and IL-6 in WT and C3H/HeJ mice following MI/R and 2 hours of reperfusion or sham operation determined with ELISA. All cytokines were significantly higher in WT mice which underwent MI/R compared to C3H/HeJ. TNF serum levels were 491.1 ± 138.6 pg/mL and 41.2 ± 9.6 pg/mL in WT MI/R and C3H/HeJ MI/R, respectively. IL-1β serum levels were 314.9 ± 76.3 pg/mL and 85.9 ± 32.2 pg/mL in WT MI/R and C3H/HeJ MI/R, respectively. Serum IL-6 was 1.6 times higher in WT (1890.6 ± 78.6 pg/mL) compared to C3H/HeJ (1178.1 ± 314.2 pg/mL). *p < 0.05 vs all groups, #p < 0.05 vs. WT sham.

We determined TNF, IL-1β and IL-6 levels in serum (table 3B). For all cytokines, significantly higher serum levels were seen in WT (n = 5) following MI/R compared to C3H/HeJ mice (n = 5, figure 5). TNF was 491.1 ± 138.6pg/mL in WT MI/R, whereas in C3H/HeJ serum level was 8.4% of WT (41.2 ± 9.6 pg/mL, p < 0.05). Similarly, IL-1β was 3.7 times higher in WT than in C3H/HeJ (314.9 ± 76.3 pg/mL vs. 85.9 ± 32.2 pg/mL, p < 0.05). Serum IL-6 was 1.6 times higher in WT MI/R (1890.6 ± 78.6 pg/mL, n = 5) compared to C3H/HeJ MI/R (1178.1 ± 314.2 pg/mL, n = 5, p < 0.05). As would be expected, for WT, all cytokines were higher with MI/R vs. sham treatment. In contrast, only IL-6 was significantly elevated in the C3H/HeJ MI/R vs. sham treatment (table 3B). Thus, in the C3H/HeJ mice, only IL-6 increased after MI/R, while TNF and IL-1β were unaffected.

## Discussion

### MI/R and Innate Immunity

Innate immunity is a rapid-response defense system, employing receptors that can recognize a broad spectrum of pathogens without prior sensitization, as is needed for acquired immunity. The innate immune system contributes to cardioprotective effects following preconditioning as well as to possible deleterious effects following MI/R. Several studies have shown that sublethal doses of LPS mediate cardioprotective effects, by attenuating activation of NFκB and reduced expression of proinflammatory cytokines [15-17]. In contrast, previous studies from our laboratory have identified TLR4 as mediating LPS-induced impairment of LV function, cardiac myocyte contractility, intramyocardial cytokine expression and NO production, as well as significant activation of myocardial NFκB, in a model of LPS induced myocardial dysfunction [18-21]. Thus, the role of TLR4 in cardiac injury is complex and not well understood.

### Findings of the present study

The most important and novel finding of the present study is that despite smaller infarcts, cardiac function was not preserved in TLR4-deficient mice. We investigated cardiac function with echocardiography and hemodynamic measurements. Both methods confirmed that cardiac function was not preserved in TLR4-deficient mice despite a smaller infarct size. We found that expression of the cardiodepressive cytokines TNF and IL-1β did not differ between TLR4-deficient and WT mice hearts. On the other hand, WT mice showed a robust expression of systemic cytokines compared to TLR4 deficient mice. Thus, the TLR4 pathway could play a more prominent role in the systemic inflammatory reaction than locally in the heart after MI/R.

In contrast to other recent MI/R studies with TLR4-deficient mice, we employed a closed-chest model, which allows cytokine levels to return to baseline levels after surgery and before initiation of ischemia [22]. Importantly, MI/R in the present study was conducted under conditions of spontaneous breathing. Mechanical ventilation in an open-chest model induces an arterial hypotension due to loss of negative intrathoracic pressure and positive end-expiratory pressure as Guo et al. have described [23].

Recently, Oyama et al. reported a reduced infarct size in TLR4-deficient mice strains compared to WT in an open-chest model, with less inflammation in the myocardium, manifested by less neutrophils, myeloperoxidase, lipid peroxides and complement deposition [8]. Our data are in good agreement with Oyama's results with respect to infarct size.

Although several studies have shown that MI/R increases myocardial TNF concentrations, which contributes to irreversible tissue injury and myocardial dysfunction [2], Chong et al. reported that TNF mRNA expression was not increased after MI/R in wild type and C3H/HeJ mice compared to sham-operated mice, whereas myocardial mRNA expression of IL-1β, monocyte chemotactic factor-1 and IL-6 was significantly increased in wild type mice [9]. Although cytokine expression is thought to be primarily regulated at the transcriptional level, post-transcriptional events can alter gene expression. Therefore, we measured the actual level of the three cytokines and found significantly higher myocardial TNF, IL-1β and IL-6 expression in WT MI/R compared to shams, which confirms other studies showing that myocardial TNF is increased during MI/R [2]. Most striking, there was no significant difference for myocardial TNF between wild type and C3H/HeJ mice after MI/R, and similar levels of protein expression were also found for IL-1β. Only myocardial **IL-6 protein expression was significantly decreased **in TLR4-deficient mice compared to WT after MI/R. This led us to the conclusion that the regulation of myocardial cytokines differs from the regulation of serum cytokines, and that at least TNF and IL-1β are not primarily regulated through TLR4 in myocardial ischemia/reperfusion.

Mechanical stress is associated with myocardial infarction triggering myocardial production of TNF and IL-6, not only in the infracted, but also in the non-infarcted region of the heart [24,25]. TNF, IL-1β and IL-6 contribute to cardiac dysfunction through activation of neutral sphingomyelinase and NO production [26,27]. However, we did not observe differences in myocardial cytokines between WT and C3H/HeJ MI/R, except for IL-6, which was significantly decreased in TLR4-deficient MI/R mice. The role of myocardial cytokines on cardiac function in the present study remains unclear. Deten et al. have suggested that IL-6 and IL-1β synergistically promote compensatory cardiac hypertrophy in the non-infarcted myocardium, [28] and other studies provide evidence that IL-6 also protects cardiac myocytes from apoptosis [29]. Numerous myocardial infarction studies have shown pleiotropic effects of these cytokines and their specific effects on cardiac function remain to be fully elucidated. It has to be considered that the absence of TLR4, not only reduces infarct size, but also other regulatory proteins, such as the chemokine monocyte chemoattractant protein (MCP)-1, which may prolong inflammation and delay replacement of injured cardiac myocytes [30].

Serum cytokines did not reflect myocardial cytokine expression, and were significantly less in TLR4-deficient mice compared to their WT littermates. Our data supports that ischemia stimulates cytokine expression via TLR4-independent mechanisms (such as mechanical stretch), resulting in similar myocardial levels of TNF and IL-1β in both strains. With the onset of reperfusion in TLR4-deficient mice, cytokine and chemokine signaling is disturbed because of the non-functional TLR4-receptor. This can result in decreased chemotaxis of leukocytes. Alteration in the inflammatory response to myocardial infarction may account for the observed discrepancy between infarct size and cardiac function for the TLR4 deficient mice.

A recent study has shown that TLR4 in cardiac myocytes mediates survival through MyD88 and NOS2 using TLR4 -/- cardiac myocytes in comparison to wild type in a model of serum deprivation induced apoptosis [10]. Other studies confirm that LPS, as a ligand of TLR4, can be cardioprotective [11-13]. It remains to be elucidated if TLR4 is essential for *in vivo *myocardial function and protection from cardiac myocyte apoptosis after MI/R. It is unclear, if TLR4 deficiency ameliorates reperfusion injury due to blunted systemic inflammatory response as shown by our present study but leads to myocardial dysfunction due to apoptosis in addition to TLR4 independent cytokine expression.

### Differences in heart rate

In this study, mice were sedated with propofol i.p. for induction of MI/R. Propofol was chosen because of its lack of preconditioning effects. However, we found a significant decrease in heart rate only in WT, probably an effect of propofol which has been also reported in C57Bl/6 mice [31]. With the onset of ischemia, heart rate increased which was attributed to stress. Interestingly, in C3H/HeJ mice propofol did not decrease heart rate. We also observed an invariable heart rate with onset of MI/R compared to WT. The mechanism underlying absence of heart rate increase despite stress of MI/R in these mice is unknown.

## Conclusion

TLR4 contributes to myocardial infarction by inducing a systemic and a myocardial cytokine response after MI/R. It has to be considered that TLR4-independent pathways are also activated due to MI/R and contribute to myocardial dysfunction. The targeted pharmacologic inhibition of TLR4 in the clinical setting of myocardial ischemia and reperfusion could initially lead to a smaller infarct, but an altered inflammatory response could have unexpected effects on cardiac function and long term infarct healing. Thus, further investigations are needed to elucidate the role of TLR4 in infarct size, modulating inflammatory responses and its impact on cardiac function.

## Methods

### Ischemia/Reperfusion experiments

A chronic, closed-chest model of MI/R was utilized in order to exclude that any inflammatory reaction following MI/R is due to the surgical trauma itself. Male, 8–12 weeks old C3H/HeJ (n = 15) and WT (C3H/HeN, n = 10; Charles River, Sulzfeld, Germany) were anesthetized with isoflurane 2.5 Vol.% (Forene^®^, Abbott GmbH, Wiesbaden, Germany) with 1 L*min^-1 ^O_2 _flow. After sufficient anesthesia depth was confirmed by squeezing the paws, animals were placed in a supine position for intubation on a water warmed plate. Body temperature and electrocardiography (ECG) were monitored and surgery was performed, as described previously [22]. The nose was placed in a nasal cone which was connected to an isoflurane vapor to maintain anesthesia during the intubation procedure. The head was slightly reclined by fixing the upper incisors with a 6-0 suture to facilitate intubation. Ventilation was confirmed by observing thorax excursions and adopted to physiological parameters (Minivent, HUGO SACHS ELEKTRONIK-HARVARD APPARATUS GmbH, March-Hugstetten, Germany, respiratory rate 105*min^-1^, 200 μl tidal volume). A left parasternal incision through the third and fourth rib was made. The chest walls were separated with retractors to achieve a good visualization of the heart. The pericardium was then gently opened and the heart was rotated on its longitudinal axis to identify the LAD with a mini tip cotton applicator (Hardwood Products Company, Guilford, Maine, USA). A U-shaped 8-0 tapered needle was cautiously passed underneath the LAD 1 mm distal from the tip of the left auricle. The 8-0 prolene suture was cut at the needle side and both ends were threaded through a 1 mm section of a PE 10 tube. Both ends of the suture were tightened to confirm the correct position of the suture by observing paleness of the distal myocardium. The following procedure was modified according to Nossuli et al. [22] who exteriorized each end of the suture with a size 3 Kalt suture needle (FST, Fine Science Tools GmbH, Heidelberg, Germany) to both sides of the thorax. Instead, we passed both ends of the suture out to the left side of the thorax, one through the second intercostal space and one through the fourth intercostal space and formed a loop by knotting each end to the other. With this modified technique we prevented a longitudinal rotation of the heart when the suture ends were pulled apart as observed with echocardiography. This rotation could potentially lead to hemodynamic changes (data not shown). The loop was then left in the subcutaneous tissue. The chest was closed with three 6-0 prolene sutures. Anesthesia was turned off while closing the skin. After mice regained spontaneous breathing they were extubated and allowed to breathe 100% O_2_.

**Figure 6:**
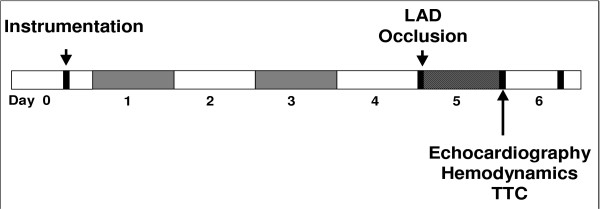
Mice were allowed to recover from instrumentation for 4 days. LAD was occluded for 60 minutes to induce myocardial infarction. Echocardiography and hemodynamic measurements were performed before hearts were excised 24 hours following ischemia for TTC staining and post-mortem analyses.

For MI/R experiments, mice were sedated with propofol i.p. (150 mg*kg^-1 ^BW), allowing spontaneous breathing, and were placed in a supine position on a warming plate beneath a heating lamp to maintain body temperature, which was monitored with a rectal probe. The ECG was monitored to document ST-segment elevation (PowerLab, ADInstruments GmbH, Spechbach, Germany). The former skin incision was reopened and after dissecting the loop, tension was carefully applied to the loop to achieve and maintain a significant ST-elevation. After 60 minutes, the loop was relaxed and it was taken care of monitoring a resolution of the ST-segment elevation as a sign of reperfusion. The loop was placed back into the subcutaneous tissue and the skin was closed. Sham-operated mice underwent the same procedure except for pulling the loop to induce myocardial ischemia/reperfusion. The time course of the MI/R protocol is summarized in figure 6. For myocardial and serum cytokine levels, animals were anesthetized following 2 hours of reperfusion, blood was collected and hearts were snap-frozen with liquid nitrogen. The specimens were stored at -80°C for further analyses. The animals were handled according to the principles of laboratory animal care (NIH publication No. 85–23, revised 1996), and animal procedures were approved by the local committee for animal care.

### Assessment of infarct size

**Figure 7:**
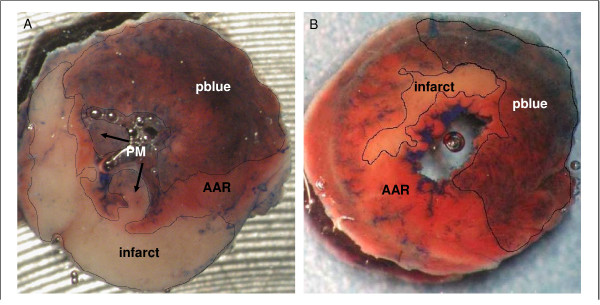
**A **Representative example of a heart slice from a WT mouse (C3H/HeN) stained with TTC. Infarct areas are not stained by TTC (white), AAR is stained red (TTC positive). Myocardium which is not perfused by LAD is stained blue by phthalo blue 10 % (pblue). Infarct involves almost all the AAR. **B **Representative example of a TTC stained myocardial slice of a TLR4-deficient mice (C3H/HeJ). IS is clearly smaller in comparison to a matchable slice of a wild type mice, pblue = phthaloblue, AAR = area at risk, PM = papillary muscle.

*In vivo*, 10% phthalo blue (Heucotech LTD, Fairless Hills, PA, USA) was injected into the left atrium 24 h following MI/R to determine the area at risk (AAR) [32]. Hearts were excised, rinsed with phosphate buffer solution (PBS) and frozen in isopentane at -160°C. Thereafter, they were cut into 7 transverse slices and incubated with 1.5% triphenyltetrazolium chloride (TTC, Sigma-Aldrich Chemie GmbH, Munich, Germany) at 37°C for 20 min in order to visualize the infarct area (figure 7). Infarct Size (IS), AAR and the phthalo blue stained areas were measured with a planimetry software by investigators unaware of sample identity (ImageJ, Version 1.29, NIH, USA). IS was calculated in percentage of the AAR.

### Echocardiographic Image Acquisition

C3H/HeJ (n = 6) and WT (n = 6) were anesthetized and heart function was assessed 24h following MI/R, as described before [33]. In brief, mice were anesthetized with sevoflurane (4% for induction, 1.5–1.8% for maintenance) in 50% nitrous oxide and 50% oxygen by facemask. Echocardiography was performed with a Philips-Ultrasound (HDI-5000) equipped with a linear-array transducer (CL15-7) operating at 15 MHz and providing frame rates up to 284 Hz. A parasternal long-axis image was used to guide perpendicular angulation of the transducer in acquisition of the short-axis slices. The most basal image was obtained by visualizing the base of the aortic root, as published recently [34]. In brief, sequenced 2D parallel short-axis images of the left ventricle were obtained in 500μm steps towards the apex by means of a micrometer-screw driven tripod. Ten to fourteen short-axis segments were recorded depending on the overall size of the left ventricle. Parasternal short-axis views were visually divided into six segments. Imaging was considered adequate when the endocardial and epicardial borders could be properly visualized in at least five segments. Cineloops of 50 frames covering minimum two heart cycles were stored digitally and analysed off-line.

### Echocardiographic Image Analysis

A practiced, single, blinded observer performed echocardiographic analyses. End-diastolic measurements were obtained at the peak of the R-wave, whereas end-systolic measurements were obtained at the time of minimum internal chamber dimensions. The acquired sequential 2D short-axis cineloops of the left ventricle were used to measure left-ventricular volumes at end-diastole and end-systole and ejection fraction (EF).

### Hemodynamic measurement of LV function

Hemodynamic measurements of LV function were obtained immediately following echocardiography after 24 h of MI/R. LV function was assessed using a 1.4-Fr Mikro-tipped Millar catheter as described previously [21]. Briefly, the right carotid artery was dissected under a microscope and a 1.4-Fr Mikro-tipped Millar catheter (Millar Instruments; Houston, Texas) was advanced into the left ventricle. The transducer was connected to a computerized data acquisition system (PowerLab, ADInstruments, Grand Junction, Colorado). The Chart 4 data analysis software (ADInstruments) was used to calculate heart rate (HR), left-ventricular developed pressure (LVDevP) and its maximal positive and negative first derivative with respect to time (+dP/dt_max_, -dP/dt_min_).

### Heart Weight

C3H/HeJ and wild type mice underwent either MI/R (n = 5) or sham (n = 6) procedure to evaluate if differences in post-MI/R myocardial edema could contribute to myocardial dysfunction. Body weights were assessed and hearts excised after 24 h of MI/R or corresponding time in sham and rinsed shortly with PBS. Left ventricular and total heart weights were determined. Afterwards, hearts were dried at 80°C for 40 min. to determine dry weights. Ratio of left-ventricular wet to dry weights as well as ratios of wet to body weight were calculated.

### Myocardial TNF, IL-1β and IL-6 protein expression

To determine TNF, IL-1β and IL-6 protein expression after 2 hours of reperfusion, snap-frozen myocardial tissue was homogenized and incubated on ice for 5 min in 1 mL of ELISA buffer containing PBS, Triton X-100 (1 μL/mL, Sigma), PMSF (250 mM in isopropanol, 1 μL/mL, Sigma, Taufkirchen, Germany) and protease inhibitors (Cat. 1836153, Roche, Mannheim, Germany). Samples were incubated on ice for 20 min, homogenized and centrifuged for 15 min at 4°C at 16,100 g. The supernatant was used for measuring myocardial TNF, IL-1β and IL-6 protein levels with ELISA (R&D Systems, Minneapolis, USA).

### Protein expression of TNF, IL-1β and IL-6 in serum

Blood was taken after 2 hours of reperfusion. Serum was assayed for levels of TNF, IL-1β and IL-6. A standard commercially available sandwich ELISA (R&D Systems, Minneapolis, USA) with a polyclonal antibody, specific for the murine recombinant cytokine, was used.

### Statistics

All values are expressed as mean ± SEM. Student's t-test was used for comparison of myocardial infarction size and cardiac function between groups. A one-way ANOVA for repeated measurements was used for heart rate. A Mann-Whitney rank sum test was used for comparison of hemodynamic values. One-way ANOVA on Ranks was used for comparison of heart weights. One-way ANOVA was used for comparison of cytokine levels followed by a Holm-Sidak pairwise multiple comparison. Differences among experimental groups were considered significant with p < 0.05.

## Authors' contributions

SK carried out the ischemia/reperfusion experiments, analyses of infarct sizes, heart weights and drafted the manuscript. AG carried out the echocardiography, hemodynamic measurements and drafted the manuscript. HS carried out the ischemia/reperfusion experiments and analyses of infarct sizes and heart weights. KT carried out analyses of echocardiographic images. PK carried out cytokine measurements. AH participated in the design of the study, interpretation of data and revised the manuscript. CG participated in the design of the study and revised the manuscript. AAK participated in the design of the study, interpretation of data, revised and helped to draft the manuscript. GB conceived of the study, and participated in its design and coordination and helped to draft the manusript. All authors read and approved the final manuscript.
